# Accuracy Standards of Wearable Technologies for Assessment of Soccer Kicking: Protocol for a Systematic Literature Review

**DOI:** 10.2196/57433

**Published:** 2024-11-04

**Authors:** Luiz H Palucci Vieira, Filipe Manuel Clemente, Felipe Armando Chang Marquez, Walter Manuel Rea Olivares, Kelly R Vargas Villafuerte, Felipe P Carpes

**Affiliations:** 1 Grupo de investigación en Tecnología aplicada a la Seguridad ocupacional, Desempeño y Calidad de vida (GiTaSyC) Facultad de Ingeniería y Arquitectura Universidad César Vallejo (UCV) Callao Peru; 2 Escola Superior Desporto e Lazer Instituto Politécnico de Viana do Castelo Viana do Castelo Portugal; 3 Sport Physical Activity and Health Research & Innovation Center Viana do Castelo Portugal; 4 Gdansk University of Physical Education and Sport Gdańsk Poland; 5 Laboratory of Neuromechanics Federal University of Pampa (Unipampa) Uruguaiana Brazil

**Keywords:** skill-related performance, shooting, team sports, sports engineering, measurement error, validity, reliability, quality control

## Abstract

**Background:**

Wearable technology is widely applied in performance monitoring, an integral part of sports and exercise sciences. The kick movement in soccer exemplifies a sports technique that could benefit from appropriate biomechanics assessment methodologies. However, the accuracy of wearables in quantifying soccer kick mechanics, particularly under field conditions, remains unclear.

**Objective:**

This study aims to present a protocol for a systematic review to discuss the measurement properties (validity, reliability, and/or accuracy aspects) of wearable technology systems explicitly used to measure ball-kicking features in soccer.

**Methods:**

This review protocol was preregistered in the Open Science Framework. A total of 2 authors will perform searches in major electronic databases using specific keyword combinations in PubMed, Physical Therapy and Sports Medicine, Web of Science, ProQuest, IEEE Xplore, EBSCOHost, and Scopus. Following a specific population, intervention, comparison, outcome framework (population: soccer players and/or collected human data in a football-related environment; intervention: at least 1 wearable used; comparator: criterion measures, repeated testing sessions and/or actual values; outcome: ball kicking data), studies will be screened based on predetermined inclusion and exclusion criteria. The methodological quality of the included studies will be assessed using the “consensus-based standards for the selection of health measurement instruments” checklist (in studies concerning validity or reliability) or the “quality assessment of diagnostic accuracy studies” tool (in studies concerning accuracy). Data extraction will be conducted to determine the level of evidence according to the “best evidence synthesis method,” and an evidence gap map will be constructed. The Cohen κ coefficient will be used to estimate the interevaluator agreement.

**Results:**

This ongoing systematic review has completed database searches and is currently in the screening phase. Depending on the number and consistency of studies, results may be presented by meta-analysis or qualitative synthesis, with subgroup analyses considering factors such as gender, age, and playing level. The final results are expected by July 2024, with manuscript submission anticipated by November 2024.

**Conclusions:**

Our study will provide a comprehensive summary of the highest level of evidence available on the use of wearables for the assessment of soccer kick mechanics, providing practical guidance for athletes and sports sciences professionals regarding the validity and reliability aspects of using wearable technology to measure ball-kicking features in soccer.

**Trial Registration:**

OSF registries https://osf.io/zm3j6

**International Registered Report Identifier (IRRID):**

DERR1-10.2196/57433

## Introduction

Soccer is well-known as one of the most practiced and watched sports around the world, with an estimated 5 billion have engaged in the last edition of the FIFA (Fédération Internationale de Football Association) World Cup Qatar 2022 [1]. A main action in soccer is the kicking. Kicking is defined as a form of striking in which the foot is used to deliver force against an object [2]. In the case of soccer, the ball is the desired object for kicking (eg, for passing or shooting). Despite the evident importance of ball kicking to soccer performance, the quantity and quality of investigations are not proportional to its relevance to the game. The lack of sufficient evidence to assist practice is evidenced, for example, by the limited number of investigations available in the form of scientific studies assessing practical interventions and their effects on ball-kicking movement [3]. While adopting field paradigms is desired when measuring or testing kicking skills in soccer [4], the difficulty of capturing player movement kinematics under field conditions may justify in part the scarcity of literature (eg, large time-frame from data collection to results report) [5-7]. In this context, the emergence of technologies that can overcome such limitations is therefore necessary.

Wearables represent a range of devices that can be worn or attached to the body to record data and comprise, in general, 2 categories: independent running devices (primary) or devices that require offline transference to a primary wearable device [8]. Wearables have been extensively used to measure load demands (internal and/or external). At the same time, this can also be used in additional sport-specific movements, such as evaluating technical aspects of gameplay [9], including but not restricted to ball kicking. Examples of wearables used to monitor soccer kicking data (eg event detection and release velocity) include inertial measurement unit [10], accelerometer [11], GPS-embedded accelerometer [12], or LPS (local positioning system) [13]. One advantage of wearable devices, which provide kinematic outputs, as compared with traditional video-based tracking techniques, refers to their generally reduced cost and time effectiveness [6].

Defining some further concepts in advance is necessary when proposing a protocol for a systematic review of the measurement properties (eg, validity and reliability) of wearables used to measure ball kicking. The first one refers to the validity term, which can be assumed as whether one given instrument measures the characteristic it was designed to quantify. Concerning kinematics variables, there is a traditional acceptance that high-speed optical systems (eg VICON; Oxford Metrics Ltd) represent the “gold standard” [14]. Reliability (eg test-retest) represents more the variation of the tool, protocol, or human system [15]. Accuracy can be defined as the closeness between a measured value and the actual value [16,17]. In the case of wearables applied to capture soccer kicking features, this can be exemplified by the number of kick occurrences registered by the device or system compared with known values derived from offline observational analysis of video recordings (ie human operator labeling). Finally, while there is a knowledge base [18-20], to the extent of our knowledge, no previous systematic reviews addressed solely the measurement properties, such as validity aspects of wearable technologies specifically applied to compute ball kicking indices.

Therefore, this study aims to provide a protocol for a systematic review focused on analyzing the measurement properties (validity, reliability, and accuracy) of wearable technology systems used explicitly to measure ball-kicking features in soccer. The future review using the protocol described here will assist in answering the following question: Are the available wearables accurate enough to quantify soccer kick mechanics, especially under field conditions?

## Methods

### Electronic Databases and Search Strategy

Searches will be performed by 2 authors (LV and FM) in the following electronic databases: (1) PubMed (National Library of Medicine), (2) Physical Therapy and Sports Medicine (Gale OneFile), (3) Web of Science (Clarivate), (4) ProQuest (ProQuest LLC), (5) IEEE Xplore (Institute of Electrical and Electronics Engineers), (6) EBSCOHost (Elton B. Stephens Company, EBSCO Information Services LLC), and (7) Scopus (Elsevier BV). No restrictions will be imposed concerning the date of publication; that is, studies will be considered for inclusion when published, even online ahead of print, from inception up to the date of searches. The searches will be updated—carried out once again—when necessary (ie, if the time interval separating literature search and manuscript acceptance for publication exceeds 12 months). The Zotero software (version 6.0.30; Corporation for Digital Scholarship, Roy Rosenzweig Center for History and New Media at George Mason University) will be used to manage reference entries from the initial search to the final inclusion steps. According to a specific Population, Intervention, Comparison, Outcome (PICO) framework [21], studies will be screened looking for those in which “P” participated able-bodied soccer players and/or collected human data in the football-related environment, “I” evaluated with at least one wearable device or system (regardless of their category, ie, commercially available or patent or research not yet commercialized), “C” that was compared against criterion measures (ie, tested validity), between testing sessions (ie, tested reliability) and/or against actual values (ie, tested accuracy) and “O” reported data for ball kicking action. Thus, search terms attempted to respect such defined PICO framework and are presented in Table 1 using a Boolean search strategy, also considering those key terms used previously in existing systematic reviews [3,14,20]. The search string formulated across the databases focused on searching using 3 major fields (title, abstract, and keywords). There was one exception of the Physical Therapy and Sports Medicine database in which a broader field of search was needed, due to a particular word limit imposed when entering the search terms in such database. After performing the last step of selection, the reference list of included studies will also be checked, aiming to identify additional studies potentially eligible for inclusion in this review.

**Table 1:** Search strategy formulated for each specific database considered for the present study protocol.

Database	Search string
PubMed	((((soccer[tiab]) OR football*[tiab]) OR association football[tiab]) OR 11-a-side[tiab]) AND (((((((((((((wearable*[tiab]) OR inertial measurement unit[tiab]) OR IMU[tiab]) OR acceleromet*[tiab]) OR microtechnology[tiab]) OR micro-electrical mechanical system[tiab]) OR MEMS[tiab]) OR global positioning system[tiab]) OR global navigation satellite system[tiab]) OR local positioning system[tiab]) OR GPS[tiab]) OR GNSS[tiab]) OR LPS[tiab]) AND (((((validity[tiab]) OR reliability[tiab]) OR measurement error[tiab]) OR accuracy[tiab]) OR precision[tiab]) AND (((((kick*[tiab]) OR shoot*[tiab]) OR pass*[tiab]) OR skill[tiab]) OR technical[tiab])
Web of Science	TS=(((((soccer) OR football*) OR association football) OR 11-a-side) AND (((((((((((((wearable*) OR inertial measurement unit) OR IMU) OR acceleromet*) OR microtechnology) OR micro-electrical mechanical system) OR MEMS) OR global positioning system) OR global navigation satellite system) OR local positioning system) OR GPS) OR GNSS) OR LPS) AND ((((((validity) OR reliability) OR measurement error) OR accuracy) OR precision) AND ((((kick*) OR shoot*) OR pass*) OR skill) OR technical))
EBSCOHost	TI ( ( soccer OR football OR association football OR 11-a-side ) AND ( wearable* OR inertial measurement unit OR IMU OR acceleromet* OR microtechnology OR micro-electrical mechanical system OR MEMS OR global positioning system OR global navigation satellite system OR local positioning system OR GPS OR GNSS OR LPS ) AND ( validity OR reliability OR measurement error OR accuracy OR precision ) AND ( kick* OR shoot* OR pass* OR skill OR technical ) ) OR AB ( ( soccer OR football OR association football OR 11-a-side ) AND ( wearable* OR inertial measurement unit OR IMU OR acceleromet* OR microtechnology OR micro-electrical mechanical system OR MEMS OR global positioning system OR global navigation satellite system OR local positioning system OR GPS OR GNSS OR LPS ) AND ( validity OR reliability OR measurement error OR accuracy OR precision ) AND ( kick* OR shoot* OR pass* OR skill OR technical ) ) OR KW ( ( soccer OR football OR association football OR 11-a-side ) AND ( wearable* OR inertial measurement unit OR IMU OR acceleromet* OR microtechnology OR micro-electrical mechanical system OR MEMS OR global positioning system OR global navigation satellite system OR local positioning system OR GPS OR GNSS OR LPS ) AND ( validity OR reliability OR measurement error OR accuracy OR precision ) AND ( kick* OR shoot* OR pass* OR skill OR technical ) )
SCOPUS	( TITLE-ABS-KEY ( soccer OR football OR “association football” OR 11-a-side ) AND TITLE-ABS-KEY ( wearable* OR “inertial measurement unit” OR imu OR acceleromet* OR microtechnology OR “micro-electrical mechanical system” OR mems OR “global positioning system” OR “global navigation satellite system” OR “local positioning system” OR gps OR gnss OR lps ) AND TITLE-ABS-KEY ( validity OR reliability OR “measurement error” OR accuracy OR precision ) AND TITLE-ABS-KEY ( kick* OR shoot* OR pass* OR skill OR technical ) )
ProQuest	TI,AB,IF(soccer OR football OR association football OR 11-a-side) AND TI,AB,IF(wearable* OR inertial measurement unit OR IMU OR acceleromet* OR microtechnology OR micro-electrical mechanical system OR MEMS OR global positioning system OR global navigation satellite system OR local positioning system OR GPS OR GNSS OR LPS) AND TI,AB,IF(validity OR reliability OR measurement error OR accuracy OR precision) AND TI,AB,IF(kick* OR shoot* OR pass* OR skill OR technical)
Physical Therapy and Sports Medicine	Entire Document: soccer OR football OR association football OR 11-a-side AND Entire Document: wearable* OR inertial measurement unit OR IMU OR acceleromet* OR microtechnology OR micro-electrical mechanical system OR MEMS OR global positioning system OR global navigation satellite system OR local positioning system OR GPS OR GNSS OR LPS AND Entire Document: validity OR reliability OR measurement error OR accuracy OR precision AND Entire Document: kick* OR shoot* OR pass* OR skill OR technical
IEEE Xplore	(“All Metadata”:soccer OR “All Metadata”:football OR “All Metadata”:association football OR “All Metadata”:11-a-side) AND (“All Metadata”:wearable* OR “All Metadata”:inertial measurement unit OR “All Metadata”:IMU OR “All Metadata”:acceleromet* OR “All Metadata”:microtechnology OR “All Metadata”:micro-electrical mechanical system OR “All Metadata”:MEMS OR “All Metadata”:global positioning system OR “All Metadata”:global navigation satellite system OR “All Metadata”:local positioning system OR “All Metadata”:GPS OR “All Metadata”:GNSS OR “All Metadata”:LPS) AND (“All Metadata”:validity OR “All Metadata”:reliability OR “All Metadata”:measurement error OR “All Metadata”:accuracy OR “All Metadata”:precision) AND (“All Metadata”:kick* OR “All Metadata”:shoot* OR “All Metadata”:pass* OR “All Metadata”:skill OR “All Metadata”:technical)

### Inclusion and Exclusion Criteria

This review will include studies presented in the form of (1) original research articles (independent of its design), (2) written in the English language, (3) published in scientific journals with peer-review policy, (4) with full-text available for download, and (5) abstract available for screening in the respective database. Suppose there is no option to obtain full-text in its original database, in that case, additional searches can be conducted in Google Scholar, allowing for potential inclusion of studies from ResearchGate (ResearchGate GmbH). As an additional inclusion criterion, (6) studies will be only considered for inclusion in the present systematic review project when they have respected the fundamental ethical principles consistent with the Declaration of Helsinki by including the full text, such information that an institutional research ethics committee approved the investigation with human subjects [22] or otherwise whether there is an explicit statement indicating that evaluations were done as a part of traditional athletes routine measurements (eg, occupationally based work or given a requirement of employees) which may allow the absence of previous approval by an appropriate body [23].

Those studies (1) examining only the application or feasibility of wearable device, (2) observations/experiments only addressing football codes movements other than ball kicking, (3) reported as grey literature, conference proceedings, books, thesis, dissertations, literature reviews, opinion pieces, case studies, (4) where a ball was not kicked, (5) with outcomes only concerning motion/flight of the ball, (6) without mention of the location where the wearable system/device was attached to the body of evaluated subjects, and (7) retracted studies will be excluded. A third researcher with long-term experience in the area (FMC) will resolve cases if no consensus is reached by the 3 authors in charge of searches/selection of studies.

### Methodological Quality and Risk of Bias Assessments

The methodological quality of the included studies will be determined using the COSMIN (COnsensus-based Standards for the selection of health Measurement Instruments) checklist, specifically the forms B (for studies assessing reliability) and H (for studies assessing validity) [24,25] or the QUADAS-2 (Quality Assessment of Diagnostic Accuracy Studies) tool (for studies assessing accuracy) [17,26]. In addition, the Risk of Bias (RoB) of results or inferences will be computed for each study included separately through the Risk of Bias Assessment Tool for Nonrandomized Studies (RoBANS; Kim et al [27]). Each item will be assessed as having low, high, or unclear risk concerning criteria of selection of participants, confounding variables, measurement of exposure, blinding of outcome assessments, incomplete outcome data, and selective outcome reporting. Review Manager software (RevMan, version 5.3; The Cochrane Collaboration) [28] will be used to construct the RoB graphs for individual studies as well as all results of all studies pooled. In addition, 2 authors (WMRO and KRVV) will perform the processes independently.

### Data Extraction and Evidence Synthesis

Data extraction will be made (by LHPV and KRVV) using a specific spreadsheet, and parameters considered to be included will be selected after piloting, with approximately 3 studies among those included. In general, we expected to extract the following data from included studies: correlation coefficients, intraclass correlation coefficient, root-mean-square error [20], F-measure, and accuracy [29] in addition to study characteristics, including, for example, device employed, time used, kick protocol, measured variable, and concluding remarks [30]. The corresponding authors of included studies will be contacted by email—those provided together with the publication—in cases where the full text lacks sufficient data or if the information presented is unclear. In the absence of a response (or negative), data will be indicated in the tables of results with the symbol “--”. Following on, the “best evidence synthesis method” will be applied to classify the level of evidence across included studies [31]; consistency has been defined when ≥75% of studies reported results in the same direction, while inconsistency when <75% of studies reported results in the same direction [3,32,33]. Thus, the following thresholds will be used:

1. Strong evidence: consistent findings obtained from multiple high-quality studies;
2. Moderate evidence: consistent findings obtained from multiple moderate-quality and/or 1 high-quality study;
3. Limited evidence: findings obtained from 1 moderate-quality and/or only low-quality studies;
4. Conflicting evidence: inconsistent findings obtained from studies;
5. No evidence: no study found.

To assist the graphical presentation of the strength of evidence, an evidence gap map will also be constructed [34,35]. For all steps where 2 evaluators are requested above (ie, literature search/selection, methodological quality/bias, and data extraction), interevaluator agreement will be assessed using Cohen κ coefficient, and then the average value across measures will be reported in the final systematic review paper.

### Ethical Considerations

The review protocol was preregistered in the Open Science Framework Registries/Generalized Systematic Review Registration (Registry ID: #ZM3J6) [36]. This protocol, as presented in full here, follows the PRISMA-P (Preferred Reporting Items for Systematic Reviews and Meta-Analyses Protocols) 2015 checklist [37] (Multimedia Appendix 1). Some updates to the original protocol registration were done based on the *JMIR Research Protocols* external reviewer’s suggestion. The projected future systematic review is also intended to contain the items proposed by the 2020 version of the PRISMA statement [38]. This study has been carried out under authorization from the Institutional Research Ethics Committee (Comité de Ética en Investigación de las Escuelas Profesionales de Educación Inicial y Ciencias del Deporte de la Universidad César Vallejo), project number 2761 (ANEXO N.º 3).

## Results

This systematic review project is ongoing. From an initial search (February 2024) conducted to find previous analyses on wearables in the context of sport (data found from inception to 2023; Table 2), there were no existing reviews, amongst those identified, which have aimed at examine solely the application of wearables to investigate ball kicking in soccer [7,9,14,18-20,39-44] (Table 3). Given the importance of the subject, a dedicated analysis is therefore justified. At the moment of submission, database searches were completed (ie, identification step), and the present authors are beginning the screening step. If there is a sufficient number of studies (ie, 3 or more on a given dependent variable) and no substantial variations are detected concerning methods used across studies, results can also be presented through a meta-analysis (quantitative synthesis). Subgroup analysis, if pertinent, will consider, for example, gender, age, and playing level. Finally, independent of whether the final manuscript will qualify for quantitative synthesis or not, qualitative synthesis will be performed using the best evidence synthesis method. In addition, a table in attachments containing the main study characteristics will be included (eg, aim, results, and findings). Results are predicted to be completed by July 2024, and the final systematic review manuscript submission by November 2024.

**Table 2:** Some methods of review studies potentially address wearable technology and soccer kicking assessment.

Reference	Type of review	Guidelines	Date of searches or inclusion	Databases considered
Adesida et al [18]	Systematic	—^a^	From inception to October 31, 2018	Scopus, MEDLINE, Embase, Cochrane Library, IEEE Xplore, Web of Science (Core Collection), and Engineering Village
Camomilla et al [19]	Systematic	PICOS^b^ framework	Until April 12, 2017	Web of Science, Scopus, PubMed, and Sport Discus
Cardinale and Varley [39]	Brief (narrative)	—	Search on July 2016	PubMed
Chambers et al [9]	Systematic	—	Published between 2008 and 2014	Academic Search Complete, CINAHL, PsycINFO, PubMed, SPORTDiscus, and Web of Science.
Crang et al [14]	Systematic	PRISMA^c^ statement	From earliest record to March 2020	SPORTDiscus, CINAHL, and MEDLINE
De Fazio et al [40]	Overview (narrative)	—	—	—
Fong and Chan [41]	Systematic	—	From 1966 to July 2010	MEDLINE, ISI Web of Knowledge (Science Citation Index Expanded), Social Sciences Citation Index, Arts & Humanities Citation Index, SportDiscus, and IEEE Xplore
Liu & Zhang [44]	Narrative	—	—	—
Lutz et al [42]	Overview (narrative)	—	—	—
Poitras et al [20]	Systematic	PRISMA	From 2005 to July 2018	PubMed, CinAHL, Ergonomic abstract, Compendex, and Embase
Rana and Mittal [7]	Narrative	—	—	—
Seçkin et al [43]	Narrative	—	Published between 2015 and 2023	Web of Science

^a^Information not reported or unclear.

^b^PICOS: Population, Intervention, Comparison, Outcome, Study design.

^c^PRISMA: Preferred Reporting Items for Systematic Reviews and Meta-Analyses.

**Table 3:** Results of an initial search for review studies potentially addressing wearable technology and soccer kicking assessment.

Reference	Year	Aim	Results related to measurement properties of wearable technologies to measure soccer kicking
Adesida et al [18]	2019	Comprehend the application of wearables in sports to optimize performance and mitigate the risk of injury	Good concurrent validity between MVN Link/Xsens inertial measurement system and Vicon, despite the higher error in segments showing fastest movements (1 study); Approximately 99% accuracy of a wearable sensor to detect kicks (1 conference proceeding); Descriptive data of application of wearables in kick investigations (2 studies)
Camomilla et al [19]	2018	Assessing the existing evidence and the prospective role of wearables in evaluating sport performance	Mention of devices assessing the accuracy of wearable sensors to detect kicks (2 studies); Descriptive data of application of wearables in kick investigations (1 study)
Cardinale and Varley [39]	2017	Review applications, obstacles, and potentials of different wearable technologies in training monitoring	—^a^
Chambers et al [9]	2015	Evaluate the use of microsensors in quantifying movements specific to sports	—
Crang et al [14]	2021	Analysis of research examining the validity/reliability of wearables in measuring actions of intermittent team sports	—
De Fazio et al [40]	2023	Offer a thorough examination of wearable technologies used for tracking the physiological aspects of patients during post-surgery recovery and athletes’ training	—
Fong and Chan [41]	2010	Examine the existing body of literature concerning the application of inertial sensors in studies focused on the biomechanics of the human lower limbs	—
Liu and Zhang [44]	2022	Provides a holistic view of recent developments in flexible/wearable sensor technologies for sports	—
Lutz et al [42]	2020	Look into contexts where wearables find application for both individual and team performance assessments, emphasizing aspects such as reliability and validity	—
Poitras et al [20]	2019	Evaluate the criterion validity and reliability of inertial measurement units according to body joint across tasks of varying levels of complexity	Mention of validity results to Xsens MVN BIOMECH (Xsens Technologies B.V., Enschede, the Netherlands) (1 study)
Rana and Mittal [7]	2021	Examining wearable technology/sensors used for performance analysis in sports	Mention of devices assessing the accuracy of wearable sensors to detect kicks (2 conference proceedings); Descriptive data of application of wearables in kick investigations (3 conference proceedings)
Seçkin et al [43]	2023	Explores the use of wearables in the measurement/monitoring of athletic components (performance, injury prevention, rehabilitation, and performance optimization)	—

^a^Not available.

## Discussion

In this study, we set out to provide a detailed protocol for a future systematic review of the measurement properties of wearables adopted to investigate ball kicking. Ensuring data quality through the integration of new technological devices is crucial for minimizing bias in research publications and the decisions derived from using such instruments in daily practice. Therefore, scientific publishing must prioritize valid metrics, as measured in concurrent-validity studies, and reliability [45]. Based on the exploratory analysis conducted in our initial search, the majority of publications indicate that wearables help quantify soccer kick mechanics. This finding would instill confidence in their use in real-world scenarios.

Recognizing the increasing demand for ecologically focused soccer drills [46] and acknowledging the opportunities presented by new technologies for incorporating measurement systems into training, the use of wearables in ecological scenarios emerges as a high-value proposition. Indeed, this approach offers a viable solution to overcome the constraints associated with gold-standard motion-based devices, which are typically limited to specific locations, predominantly within laboratories.

With the progress in microsensors, we anticipate observing satisfactory levels of concurrent validity in wearable inertial measurement units, as supported by previous original research [47,48]. The majority of kinematic measures exhibiting acceptable concurrent validity and reliability are expected to be associated with ball release velocity [48], representing an outcome of the kicking process. Meanwhile, other outcomes will be linked to the inherent quality of movement, seeking to characterize the kinematic profile of players [12,47] by measuring linear, angular, or joint velocities. It is important to note that the judgment on whether individual wearable types/brands present sufficient research evidence to be used in practice will be based on both the quality and quantity of published studies available in the form of scientific articles, as per the rigorous synthesis method adopted.

The reliability levels observed in the wearable microsensors, as mentioned above, may enable ecological-based research and practical scenarios. Despite their accuracy and precision being comparable to gold-standard vision-based instruments, the latter are, in general, significantly limited in their ability to track kinematics in confined spaces, thereby failing to provide context and real-world applicability to practice.

As a limitation, this study focuses solely on the kicking process, thereby excluding the examination of other wearable technologies that are currently being observed for upper limb movements and throws. However, it is conceivable that these technologies may become applicable to kicking in the near future. Furthermore, the limited number of articles identified in this research field, coupled with its niche nature, may constrain the generalization of findings. Nonetheless, our study addresses a specific research and practice issue within one of the most widely practiced sports globally. Consequently, it has the potential to assist provide insights into the quality of data derived from these devices for numerous coaches.

The outcomes of this systematic review will offer researchers and practitioners a means to comprehensively summarize the evidence regarding the quality of data extracted from wearable devices for measuring kicking performance. This will enable the identification of the most accurate and reliable devices, pinpoint gaps in current research, and identify new research directions and developments that need attention in the coming years. From a practical standpoint, coaches and sports scientists can confidently use wearable technology to analyze the kinematics of kicking. Such analyses can inform the design of tasks aimed at enhancing kicking performance while also providing valuable feedback to soccer athletes to improve this skill.

### Conclusions

To our knowledge, this will be the first systematic review of scientific literature to attempt to collate knowledge derived from peer-reviewed articles that covered the measurement properties related to the use of wearable devices to capture ball-kicking features. A systematic review of scientific studies addressing this issue will potentially help, for example, to (1) highlight which instruments are effective in day-to-day testing or monitoring of kicking performance, (2) to understand the potential sources of variability (eg, specific environmental properties), and (3) to clarify the operator attitudes (eg, configurations) that could be adopted as a way of capturing adequately the event of interest – ball kicking – which itself is not a trivial task. Thus, it is possible to conclude that the protocol proposed here will benefit practitioners and football scientists, together with the final results from the systematic review with the best evidence synthesis.

## Appendix

Multimedia Appendix 1 PRISMA-P (Preferred Reporting Items for Systematic Reviews and Meta-Analyses Protocols) checklist for the present systematic review protocol.

## Abbreviations

COSMIN: COnsensus-based Standards for the selection of health Measurement Instruments
FIFA: Fédération Internationale de Football Association
GPS: global positioning system
LPS: local positioning system
PICO: Population, Intervention, Comparison, Outcome
PRISMA-P: Preferred Reporting Items for Systematic Reviews and Meta-Analyses Protocols
QUADAS-2: Quality Assessment of Diagnostic Accuracy Studies
RoB: Risk of Bias
RoBANS: Risk of Bias Assessment Tool for Nonrandomized Studies

## Data Availability

Data sharing is not applicable to this article as no datasets were generated or analyzed during this study protocol. A brief literature overview is presented and all data reported are from previous published studies, which have been cited and included in the reference list.
